# Evaluation of the Performance of OTOPLAN-Based Cochlear Implant Electrode Array Selection: A Retrospective Study

**DOI:** 10.3390/jpm13081276

**Published:** 2023-08-20

**Authors:** Dayse Távora-Vieira, Marcus Voola, Jafri Kuthubutheen, Peter Friedland, Daren Gibson, Aanand Acharya

**Affiliations:** 1 Fiona Stanley Fremantle Hospitals Group, Perth, WA 6150, Australia; 2 Medical School, Division of Surgery, The University of Western Australia, Perth, WA 6010, Australia; 3 Faculty of Health Sciences, School of Allied Health, Curtin University, Perth, WA 6102, Australia; 4 Sir Charles Gairdner Hospital, Perth, WA 6009, Australia

**Keywords:** cochlear implant, OTOPLAN, electrode array, pre-operative planning, hearing outcomes, anatomy-based fitting

## Abstract

Otoplan is a surgical planning software designed to assist with cochlear implant surgery. One of its outputs is a recommendation of electrode array type based on imaging parameters. In this retrospective study, we evaluated the differences in auditory outcomes between patients who were implanted with arrays corresponding to those recommended by the Otoplan software versus those in which the array selection differed from the Otoplan recommendation. Pre-operative CT images from 114 patients were imported into the software, and array recommendations were generated. These were compared to the arrays which had actually been implanted during surgery, both in terms of array type and length. As recommended, 47% of patients received the same array, 34% received a shorter array, and 18% received a longer array. For reasons relating to structure and hearing preservation, 83% received the more flexible arrays. Those who received stiffer arrays had cochlear malformations or ossification. A negative, although non-statistically significant correlation was observed between the CNC scores at 12 months and the absolute value of the difference between recommended array and implanted array. In conclusion, clinicians may be slightly biased toward shorter electrode arrays due to their perceived greater ability to achieve full insertion. Using 3D imaging during the pre-operative planning may improve clinicians’ confidence to implant longer electrode arrays, where appropriate, to achieve optimum hearing outcomes.

## 1. Introduction

Cochlear implantation is the current gold standard treatment for severe-to-profound sensorineural hearing loss. A cochlear implant (CI) operates by directly stimulating the auditory nerve via an electrode array inserted into the cochlea. Given the tonotopic organisation of the cochlea, it is considered important that the electrode array length corresponds to the cochlear duct length (CDL) to enable the entire cochlea to be stimulated [1,2,3]. It has been known, however, since 1930 that no two cochleae have the exact same anatomical dimensions. Past studies found that the CDL typically ranges between 25.26 mm and 35.45 mm [4,5,6]. Historically, a single-length electrode array was considered to be adequate as the majority of patients were thought to have similarly sized cochleae. However, with the introduction of hearing preservation techniques and the growing awareness that array length may affect CI outcomes [7], a greater emphasis should be placed on the importance of selecting the most appropriate array length to provide optimal outcomes for the user.

Arrays that are short relative to the CDL can have a detrimental impact on the users’ hearing outcomes, leading to reduced performance in speech understanding tests [8,9,10]. On the other hand, insertion of relatively long arrays can result in trauma to the cochlear structures, which may precipitate post-operative complications such as a loss of residual hearing [11]. This is exemplified in cases of combined electric–acoustic stimulation (EAS) in patients with severe high-frequency sensorineural hearing loss. Here, full cochlear coverage is not needed due to the presence of residual low-frequency hearing. The partial coverage of the cochlea enables the high-frequency portion to be stimulated using the CI and the low-frequency section of the cochlea to be stimulated via standard acoustic stimulation [12,13,14,15]. In the last decade, electrode arrays of varying length, size, and stiffness have been introduced with the aim of facilitating individualised array selection and improving post-operative outcomes.

The methods used to estimate the CDL have evolved over time. The first method involved measuring the organ of Corti using histological sections with a micrometre [6]. This technique, however, depended on the angle at which histological sections were cut, which then had a large effect on the CDL value obtained [16]. With the development of computed tomography (CT), spiral models could be used to estimate the CDL based on different cochlear basal turn dimensions. However, this method was based on the assumption that all patients had cochleae of the same shape which is now known to be incorrect [6,17]. More recently, a method was developed that uses 3D reconstruction of the cochlear structures from CT scans [18].

In 2018, an otological planning software was developed for robotic CI implantation. The software can estimate the CDL and the corresponding frequency map, as well visualising the projected location and coverage of different array types [18]. This software reconstructs the cochlea in 3D from conventional CT based on its diameter, width, and height, which are measured manually by the clinician. The software provides clinicians with an estimate of the length of every turn and of the frequency range coverage of a given electrode array, referenced to the audiogram of each patient. In doing so, it can provide guidance in optimal electrode array selection.

This software has been validated as a useful and reliable tool for estimating CDL, angular insertion depth (AID), and frequency mapping [19,20], and is gradually entering into routine clinical practice [21,22]. Chen et al. [23] described it as faster and more reliable for cochlear measurement than traditional curved multiplanar reconstruction. Lovato and colleagues argue that it can guide array choice and help avoid incomplete insertion for ossified and malformed cochleae [24,25]. Many CI surgeons, however, continue to use their clinical judgment for array selection. This judgment is based on their experience with electrode arrays that are considered one-size-fits-all. Additionally, surgeons opt for traditional shorter electrode arrays for EAS scenarios.

The primary aim of this study was to evaluate to what extent the *clinician-based choice* of an electrode array differs from the *3D-imaging-based choice* for a specific electrode array length and the respective outcomes. The secondary aim of the study was to evaluate if the auditory performance differed between cases where the selected electrode was or was not in agreement with the *3D-imaging-based choice* in terms of array length.

## 2. Materials and Methods

### 2.1. Participants

For this retrospective study, we examined records of patients with bilateral hearing loss (BiHL) or single-sided deafness (SSD) who had received a MED-EL CI (MED-EL GmbH, Innsbruck, Austria) at a tertiary hospital in Western Australia between 2013 and 2020. Participants were included in the study if they were unilateral or bilateral CI users, had profound sensorineural hearing loss of any aetiology and duration, and had had high-resolution CT scans of the temporal bones as part of the pre-operative planning. Participants were excluded if their pre-operative CT images were not available at the time of review. The following information was obtained from the participants’ medical records: demographic information, the type of electrode array implanted, the reason for array selection (if documented), and pre- and post-operative audiometric test results. This study was designed and conducted in accordance with the Declaration of Helsinki, and ethical approval was obtained from the South Metropolitan Area Health Service Human Research Ethics Committee, Australia.

### 2.2. Speech Perception Tests

Speech perception tests were conducted in quiet and in noise in a soundproof booth with the loudspeakers placed at ear level, 1 m from the centre of the subject’s head. For participants with BiHL, aided speech perception was tested in quiet using the consonant–nucleus–consonant (CNC) test, with the stimuli presented at 65 dB SPL. Pre-operative aided testing was performed using a hearing aid and post-operative testing was conducted using a CI. Participants with SSD underwent a speech perception in noise test under three spatial configurations: speech and noise presented from the front (S_0_N_0_), speech from the front and noise from the hearing ear (S_0_N_HE_), and speech from the CI side and noise from the hearing ear (S_CI_N_HE_). The Bamford–Kowal–Bench speech-in-noise (BKB-SIN) test was used with an adaptive procedure to measure the signal-to-noise ratio required to achieve an intelligibility score of 50% (SRT_50_).

### 2.3. 3D-Imaging

Pre-operative CT images were imported from the hospital’s picture archiving and communication system into the OTOPLAN software (version 3, CAScination AG, Bern, Switzerland and MED-EL GmbH, Innsbruck, Austria) to create a 3D reconstruction of the cochleae. A clinician then manually marked key anatomical cochlear structures such as the modiolus, round window, apical, and basal turns. A single clinician marked all scans in order to avoid any inter-evaluator variability. These marks are used by the software to automatically calculate cochlear diameter, width, height, and CDL. Based on the measurements and visualisation of the CDL in relation to the audiogram, the clinician selected one of the FLEX electrodes arrays that would be the best fit for the given cochlea. The following electrode arrays for selection were as follows: FLEX^20^, FLEX^24^, FLEX^26^, FLEX^28^, or FLEX^soft^, where the array length in mm is given in superscript (the FLEX^soft^ array has a length of 31.5 mm). The 3D imaging software estimated the AID and frequency that would be reached by the selected array. This electrode array selection is referred to throughout the manuscript as the *3D-imaging-based choice*, whilst the implanted electrode array is referred to as the *clinician-based choice*.

The AID and frequency at the most apical electrode contact (C1) were also calculated for the implanted array in the 3D imaging software. The data were exported into a Microsoft Excel file.

### 2.4. Data Analysis

The data were analysed in IBM SPSS Statistics Version 24 (IBM, Armonk, NY, USA). Descriptive summary statistics including mean, median, standard deviation and range, were calculated for demographic, electrode array, and speech test data. Speech test data were divided into three subgroups: a group that received an electrode array that coincided with the *3D-imaging-based choice*, a group that received a shorter array, and a group that received a longer array. The Kruskal–Wallis H-test was used to assess the difference in performance between the three groups at different time points (pre- and post-operatively). The Mann–Whitney U-test was used for pairwise group comparisons. Results were considered statistically significant at *p* values < 0.05.

## 3. Results

### 3.1. Participants

A total of 114 adult unilateral CI users were included in this study with 66 (57.9%) being male, and 48 (42.1%) being female. Of these, 49 (43.0%) had SSD and 65 (57.0%) had BiHL whilst 67 (58.8%) had a CI on the right ear and 47 (41.2%) on the left ear. In the SSD group, the mean time since the onset of hearing loss was 10.0 years (range: 0.1–41.0 years) and the mean age at implantation was 50.5 years (range: 1.3–79.4 years). In the BiHL group, the mean time since the onset of hearing loss was 2.2 years (range: 0.04–8.0 years), the mean age at implantation was 61.4 years (range: 18.4–85.0 years), and the mean time since implantation was 5.3 years (range: 0.8–11.7 years).

### 3.2. Electrode Array Implanted versus the 3D Imaging Recommendation

Of the participants, 54 (47.4%) received the same electrode array as was recommended by the 3D imaging software, while 39 (34.2%) received a shorter electrode array and 21 (18.4%) received a longer electrode array. FLEX^28^, the second-longest implanted array, was both the most commonly recommended (47.4%) and the most commonly implanted (65.8%). FLEX^soft^, the longest implanted array, was also commonly recommended using the 3D imaging software (42.1%), although it was implanted in only 5.3% of cases. The numbers of implanted and recommended electrode arrays of each type are given in Table 1.

We reviewed the clinical case notes recorded at the time of insertion to determine the reasons the implanting surgeon chose the electrode. Longer electrode arrays were implanted for one of the following three reasons: (1) to provide a possible wider range of electrical stimulation if any residual hearing deteriorates over time, (2) due to the range of electrode arrays available at the time of surgery, or (3) due to the surgeon’s preference for a deeper insertion. Shorter electrode arrays were implanted for one of two reasons: (1) due to the surgeon’s preference for a shorter electrode array, and (2) due to the presence of cochlear abnormalities such as ossification or fibrosis.

Of the participants, 95 (83.3%) received a soft electrode array from the FLEX series (FLEX^24^, FLEX^26^, FLEX^28^, or FLEX^soft^) whereas, the remaining 19 (16.7%) received a stiffer electrode array (FORM^19^, FORM^24^, MEDIUM (24 mm), or STANDARD (31.5 mm)).

### 3.3. Cochlear Duct Length and Angular Insertion Depth

The mean CDL was 34.4 mm in the group that received an array corresponding to the *3D-imaging-based choice*. The mean CDL was 36.2 mm in the group that received shorter arrays, and 33.5 mm in the group that received longer arrays (Table 2).

The predicted AID and the frequency reached by the most apical electrode contact (C1), obtained using the 3D imaging software, were compared with the actual values obtained post-operatively (Table 2). The predicted and actual mean AID and frequency values were the same in the group that received the recommended arrays (mean C1 angle: 611.8°; mean C1 frequency: 230.6 Hz). The mean C1 angle was smaller (511.4° vs. 637.1°) and the mean C1 frequency was higher (433.9 Hz vs. 181.5 Hz) in the group that received shorter electrode arrays. Conversely, the mean C1 angle was larger (668.4° vs. 523.9°) and the mean C1 frequency was lower (223.5 Hz vs. 493.0 Hz) in the group that received longer electrode arrays.

### 3.4. Speech-in-Quiet Tests in the BiHL Group

**Figure 1:**
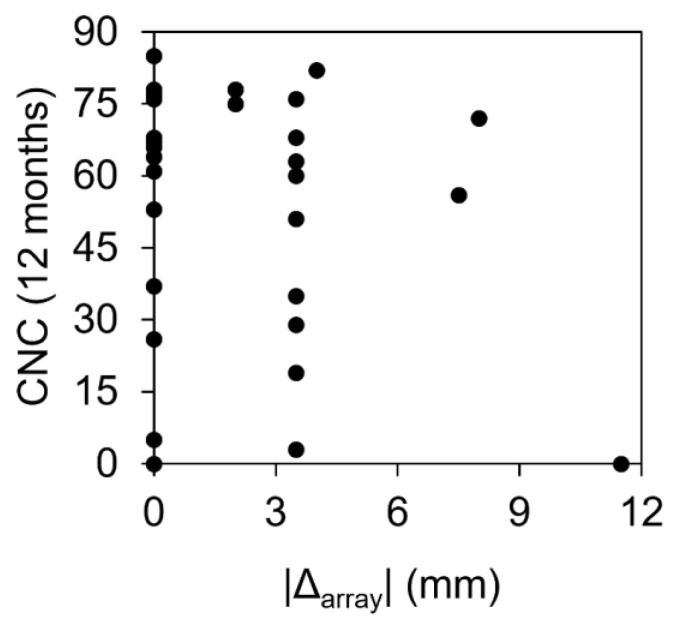
Speech-in-quiet (CNC) scores plotted against the absolute difference in electrode length between the *3D-imaging-based choice* and the implanted array.

The BiHL group underwent CNC word tests pre- and post-operatively. Subgroup averages were calculated separately for those who received the same arrays, longer arrays, and shorter arrays (Table 3). The Kruskal–Wallis H-test did not reveal any significant differences between the three groups at either time point. Further analysis of the data was performed on the basis of the absolute difference in electrode length between the recommended array and the implanted array. Figure 1 shows the CNC scores at 12 months plotted against the absolute value of the difference between the recommended array and implanted array. A negative Pearson correlation (−0.23) was observed, but this correlation was not significant (*p* = 0.2).

### 3.5. Speech-in-Noise Tests in the SSD Group

**Figure 2:**
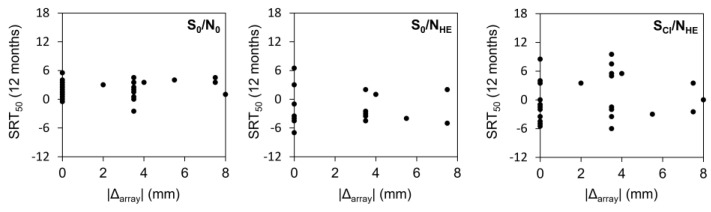
Distributions of SRT_50_ values by the absolute difference in array length between the *3D-imaging-based choice* and the implanted arrays. Data are shown separately for the three different speech-in-noise listening conditions.

The SSD group underwent speech-in-noise tests (S_0_N_0_, S_0_N_HE_, S_CI_N_HE_) pre- and post-operatively (Figure 2). Subgroup averages were calculated separately for those who received the same arrays, longer arrays, and shorter arrays (Table 4). The Kruskal–Wallis H-test did not reveal any significant differences between the group that received the arrays recommended by the 3D imaging software and the group that received shorter arrays. The group with longer arrays was not included in the analysis because insufficient data were available to provide a valid statistical comparison.

## 4. Discussion

The primary aim of this study was to retrospectively compare the differences between the arrays recommended using the 3D imaging measurement based on pre-operative CT images (*3D-imaging-base choice*) and the electrode arrays chosen by clinicians at the time of surgery (*clinician-based choice*). The secondary aim was to determine if there was a difference in the auditory performance of patients who had an implanted array that was the same length as, shorter, or longer than the *3D-imaging-based choice*.

Our findings indicate that, out of 114 implanted arrays, 47% matched the *3D-imaging-based choice*, suggesting that 3D imaging software supported the expert opinion of the clinician in just under half of cases. Clinicians were almost twice as likely to choose a shorter (34%) than a longer (18%) electrode array relative to the *3D-imaging-based choice*. This result suggests that clinicians tend to favour selecting the electrode arrays that would, in their opinion, be most likely to fit the majority of cochleae irrespective of their CDL. For example, 30 (34%) participants could have received the longest FLEX^soft^ array (31.5 mm) but instead received the FLEX^28^ (28 mm) array. In this group, CNC scores at 12 months were lower compared to those that were implanted with longer arrays. It is possible that if the 3D imaging software had been used during pre-operative planning, a longer array could have been recommended and implanted. When clinicians implanted an array longer than that recommended, the cited reasons were either electrode array availability at the time of surgery, or concerns about long-term hearing outcomes when using a shorter array if residual hearing deteriorated.

The choice of electrode array by most clinicians is typically informed by the evidence that longer arrays are associated with better auditory performance, but worse hearing preservation [13,26]. This is ostensibly because deeper insertions are considered to be associated with higher levels of damage to the cochlear structures [27]. However, hearing preservation rates vary, even when the same electrode arrays are used, because residual hearing is associated not only with the array length but also with natural disease progression and additional factors other than electrode design [13,28,29]. It is generally accepted that hearing can be preserved even when longer arrays are used, albeit to a lesser extent [28,30].

In our study, arrays differed not only in length, but also in stiffness. We included three electrode array families: FLEX, FORM, and CLASSIC. FLEX arrays are soft, flexible, and have a thinned tip. These features are designed to make cochlear implantation as atraumatic as possible, with the aim of maximising hearing preservation. FORM and CLASSIC (STANDARD and MEDIUM) arrays are stiffer than FLEX arrays and are designed specifically for malformed or ossified cochleae. In our study, 83% of participants received a FLEX array. Stiffer arrays were only implanted in malformed or ossified cochleae, in accordance with the manufacturer’s guidelines. This distribution of array selections suggests that in our centre, when selecting from a range of electrode arrays, clinicians preferred a less traumatic array for structure and hearing preservation and that, in the majority of patients with normal cochlear anatomy, it is not necessary to utilise stiff arrays [30].

The mean CDL was 34.4 mm in the group that received the same arrays of the same length as the *3D-imaging-based choice*, 36.2 mm in the group that received shorter arrays, and 33.5 mm in the group that received longer arrays. These mean values correspond to the higher end of the CDL range reported in the literature [4,5,6]. This difference may be related to our specific population of patients. As expected, the AID was greater and the C1 frequency was lower when the implanted array was longer than the *3D-imaging-based choice*; the opposite was true for shorter arrays. This suggests that the length of the electrode array has a measurable effect on the place pitch matching. This contrasts with the previously held idea that a one-size-fits-all approach to electrode array selection is sufficient.

We attempted to ascertain whether diverging from the *3D-imaging-based choice* for array selection has an effect on post-operative speech outcomes. A negative Pearson correlation (−0.23) was observed between speech-in-quiet scores at 12 months and the absolute value of the difference between the recommended array and implanted array, but this did not rise to the level of statistical significance (*p* = 0.2). This finding contrasts with those of previous studies that measured speech performance as a function of cochlear place-to-frequency mismatch, analogous to the use of shorter or longer arrays than appropriate for a given CDL. Mertens et al. [31] reported a significant correlation between frequency-to-place mismatch and speech perception in noise at 6 months of CI experience, but not at 12 months. Canfarotta et al. [10,32] similarly reported that CNC speech perception scores at 1, 3, and 6 months of CI experience correlated negatively with increasing frequency-to-place mismatch, but this effect was largely age-dependent, with older users being less affected. The same 3D imaging software was employed in these studies to measure frequency–place mismatches.

Relative to the previous studies, our study analysed speech perception at a later timepoint (12 months). This may plausibly explain the divergence between our findings and those of the previous studies. It is possible that neuroplastic effects may allow the user to gradually accommodate to a frequency–place mismatch, eventually reaching speech perception abilities equivalent to those of users without a mismatch. It nevertheless remains the case that the presence of a mismatch deprives the user of optimal hearing outcomes for a duration of several months post-activation. This is undesirable in and of itself, even if the deficit resolves over time. Furthermore, such deprivation might leave subtle long-term or permanent deficits that are not revealed by simple speech-in-quiet or speech-in-noise testing.

As noted, cochlear anatomy and residual hearing can vary greatly between individuals. Variation in CDL, anatomical anomalies, and the proportion of surviving hair cells means that one size cannot simply fit all. Specialised arrays of different length and stiffness can ensure both full insertion and complete coverage of the cochlear duct in all types of cochleae. The technique known as 3D imaging is designed to facilitate electrode array selection based on individual cochleae parameters and is gradually gaining validity in CI surgery planning [19,20,21,22,24,25]. Overall, the results of this study highlight a discrepancy between the electrode arrays that clinicians choose to implant and those arrays that could be implanted if 3D imaging software were routinely used in pre-operative planning. The 3D imaging software could incentivise clinicians to opt for longer arrays and potentially lead to better hearing outcomes in cases where clinicians would otherwise select shorter arrays.

Limitations of this study: it is important to note that our study was not powered to reliably assess the association between array choice and auditory outcomes, so our results are preliminary, and analysis of larger test groups is required. Additionally, the retrospective nature of this study is an important limitation, and it would be interesting to replicate these findings in a prospective multi-centre design, controlling for other variables that could have enhanced the difference between the groups.

## 5. Conclusions

Clinicians preferred an electrode array that differed from the 3D imaging software recommendation in approximately 50% of cases. Shorter and more flexible arrays were favoured for their perceived benefits in structure and hearing preservation. Routine 3D imaging software use would lead to greater longer electrode implantation, with the potential to achieve better hearing outcomes.

## Figures and Tables

**Table 1:** The distribution of implanted electrode arrays versus the *3D-imaging-based choice*.

	*3D-Imaging-Based Choice*	Implanted Array								
*Relative Length*		FLEX^24^	FLEX^26^	FLEX^28^	FLEX^soft^	FORM^19^	FORM^24^	MEDIUM	STANDARD	Total (%)
Same	FLEX^28^			41						41
	FLEX^soft^				6				7	13
	**Subtotal**			41	6				7	**54 (47.4)**
Shorter ^a^	FLEX^28^	1				1	2			4
	FLEX^soft^	2	1	30			1	1		35
	**Subtotal**	3	1	30		1	3	1		**39 (34.2)**
Longer ^b^	FLEX^20^	2	3						1	6
	FLEX^24^		1							1
	FLEX^26^		4	1						5
	FLEX^28^			3					6	9
	**Subtotal**	2	8	4					7	**21 (18.4)**
	Total	5	9	75	6	1	3	1	14	114

^a^ Shorter—implanted array was shorter than the *3D-imaging-based choice*; ^b^ Longer—implanted array was longer than the *3D-imaging-based choice*.

**Table 2:** Cochlear duct length, angular insertion depth and reached frequency (implanted and *3D-imaging-based choice*).

Relative Length (n)	Statistic	CDL (mm)	AID (Deg): *3D-Imaging-Based Choice*	AID (Deg): Implanted	C1 Frequency (Hz): *3D-Imaging-Based Choice*	C1 Frequency (Hz): Implanted
Same (54)	Mean	34.4	611.8	611.8	230.6	230.6
	Median	34.2	605.3	605.3	227.6	227.6
	SD	1.7	28.8	28.8	78.1	78.1
	Range	31.6–39.6	566.3–680.0	566.3–680.0	124.6–562.7	124.6–562.7
Shorter ^a^ (39)	Mean	36.2	637.1	511.4	181.5	433.9
	Median	36.1	635.1	532.6	180.0	360.7
	SD	1.4	35.1	62.8	47.8	200.6
	Range	33.3–38.8	576.8–734.1	304.5–612.7	74.9–272.5	211.8–1311.7
Longer ^b^ (21)	Mean	33.5	523.9	668.4	493.0	223.5
	Median	33.6	593.2	695.4	418.8	115.8
	SD	2.3	119.2	112.8	355.2	202.2
	Range	27.9–38.3	315.7–655.0	459.5–851.7	153.0–1235.2	7.0–631.6

^a^ Shorter—implanted array was shorter than the *3D-imaging-based choice*; ^b^ Longer—implanted array was longer than *3D-imaging-based choice*. AID—angular insertion depth in degrees, C1 frequency—frequency at the most apical electrode contact, CDL—cochlear duct length, SD—standard deviation.

**Table 3:** BiHL CNC speech test—subgroup averages.

Relative Length	Statistic	Pre-op CNC	12-Month CNC
		IE Aided	CI Alone
Same	N	16	15
	Mean	6.9	55.9
	Median	0.0	66.0
	SD	18.2	26.8
	Range	0–72	0–85
Shorter ^a^	N	13	9
	Mean	10.4	42.7
	Median	0.0	51.0
	SD	18.0	22.3
	Range	0–53	3–68
Longer ^b^	N	7	6
	Mean	21.7	63.8
	Median	0.0	75.5
	SD	28.9	31.4
	Range	0–65	0–82

^a^ Shorter—implanted array was shorter than the *3D-imaging-based choice*; ^b^ Longer—implanted array was longer than the *3D-imaging-based choice*. BiHL—bilateral hearing loss group, CNC—consonant–nucleus–consonant, IE—ipsilateral ear, SD—standard deviation.

**Table 4:** SSD speech-in-noise test—subgroup averages.

Relative Length	Statistic	S_0_N_0_		S_0_N_HE_		S_CI_N_HE_	
		Pre-op	12-Month	Pre-op	12-Month	Pre-op	12-Month
Same	N	7	7	5	5	7	7
	Mean	4.6	2.4	0.0	−1.8	5.6	−0.2
	Median	4.0	2.0	−1.0	−2.5	4.0	−0.5
	SD	2.2	2.1	3.7	3.3	3.5	3.0
	Range	1.5–8.5	0.0–5.0	−5.0–5.0	−5.0–3.0	2.0–10.5	−3.5–5.0
Shorter ^a^	N	7	7	3	3	7	7
	Mean	4.6	2.7	−0.7	−0.5	7.1	2.8
	Median	4.5	3.0	−1.0	0.5	9.0	2.0
	SD	1.7	2.3	3.0	2.6	2.5	5.1
	Range	2.5–6.5	−2.0–5.0	−3.5–2.5	−3.5–1.5	3.0–9.0	−2.0–12.0
Longer ^b^	N	3	3	1	1	3	3
	Mean	4.0	2.7	−3.5	−2.5	2.5	−1.7
	Median	4.5	2.5	−3.5	−2.5	2.0	−3.5
	SD	1.3	1.3	−	−	2.3	3.6
	Range	2.5–5.0	1.5–4.0	−	−	0.5–5.0	−4.0–2.5

^a^ Shorter—implanted array was shorter than *OTOPLAN-based choice*; ^b^ Longer—implanted array was longer than *OTOPLAN-based choice*. CI—cochlear implant, HE—hearing ear, SD—standard deviation, SSD—single-sided deafness.

## Data Availability

Due to the nature of the medical records analysed here, the underlying data cannot be publicly shared.
